# Joint estimation of causal effects from observational and intervention gene expression data

**DOI:** 10.1186/1752-0509-7-111

**Published:** 2013-10-31

**Authors:** Andrea Rau, Florence Jaffrézic, Grégory Nuel

**Affiliations:** 1 INRA, UMR1313 Génétique animale et biologie intégrative, 78352 Jouy-en-Josas, France; 2 AgroParisTech, UMR1313 Génétique animale et biologie intégrative, 75231 Paris 05, France; 3 MAP5, UMR CNRS 8145, University Paris Descartes, 45 rue des Saints-Pères, F-75006 Paris, France; 4 Sorbonne Paris Cité, Paris, France

**Keywords:** Causal inference, Gaussian Bayesian network, Intervention calculus, Metropolis-Hastings, Maximum likelihood

## Abstract

**Background:**

In recent years, there has been great interest in using transcriptomic data to infer gene regulatory networks. For the time being, methodological development in this area has primarily made use of graphical Gaussian models for observational wild-type data, resulting in undirected graphs that are not able to accurately highlight causal relationships among genes. In the present work, we seek to improve the estimation of causal effects among genes by jointly modeling observational transcriptomic data with arbitrarily complex intervention data obtained by performing partial, single, or multiple gene knock-outs or knock-downs.

**Results:**

Using the framework of causal Gaussian Bayesian networks, we propose a Markov chain Monte Carlo algorithm with a Mallows proposal model and analytical likelihood maximization to sample from the posterior distribution of causal node orderings, and in turn, to estimate causal effects. The main advantage of the proposed algorithm over previously proposed methods is its flexibility to accommodate any kind of intervention design, including partial or multiple knock-out experiments. Using simulated data as well as data from the Dialogue for Reverse Engineering Assessments and Methods (DREAM) 2007 challenge, the proposed method was compared to two alternative approaches: one requiring a complete, single knock-out design, and one able to model only observational data.

**Conclusions:**

The proposed algorithm was found to perform as well as, and in most cases better, than the alternative methods in terms of accuracy for the estimation of causal effects. In addition, multiple knock-outs proved to contribute valuable additional information compared to single knock-outs. Finally, the simulation study confirmed that it is not possible to estimate the causal ordering of genes from observational data alone. In all cases, we found that the inclusion of intervention experiments enabled more accurate estimation of causal regulatory relationships than the use of wild-type data alone.

## Background

The inference of gene regulatory networks from transcriptomic data has been a wide research area in recent years. Several approaches have been proposed to infer networks from observational transcriptomic data (also referred to as wild-type or steady-state expression data), mainly based on the use of graphical Gaussian models [1]. These methods, however, rely on the estimation of partial correlations and result in undirected graphs that cannot highlight the causal relationships among genes. For this reason, a great deal of research has focused instead on the use of causal Bayesian networks for a wide variety of applications [2,3].

As an example, [4] and [5] make use of causal Bayesian networks in the case of multinomial data, where the former applies a score-based method and the latter samples graph structures using a Markov chain Monte Carlo (MCMC) approach. Using Gaussian causal Bayesian networks (GBN), Maathuis *et al.*[6,7] recently proposed a method called *Intervention-calculus when the DAG is Absent* (IDA) to predict bounds for causal effects from observational data alone. In the IDA, the PC-algorithm [2,8,9] is first applied to find the associated completed partially directed acyclic graph (CPDAG), corresponding to the graphs belonging to the appropriate equivalence class. Following this step, bounds for total causal effects of each gene on the others are estimated using intervention calculus [10] for each directed acyclic graph (DAG) in the equivalence class.

However, if intervention experiments such as gene knock-outs or knock-downs are available, it is valuable to jointly perform causal network inference from a combination of wild-type and intervention data. One such approach has been proposed by Pinna *et al.*[11], based on the simple idea of calculating the deviation between observed gene expression values and the expression under each systematic intervention. In particular, Pinna *et al.* propose the calculation of several matrices to evaluate the differences between observational and intervention expression values: a simple deviation matrix, a standardized deviation matrix, and a z-score deviation matrix. In addition, for large networks (e.g., 100 genes), a down-ranking algorithm is applied to the initial graph obtained from these deviation matrices to remove feed-forward edges. In order to evaluate all possible causal links among genes, the method requires a single replicate of observational data as well as a single knock-out experiment for each gene in the network. An improved version of the Pinna approach was very recently proposed [12] to provide more accurate network inference for large-scale networks through a novel implemention of the transitive reduction step. As with the originally proposed method, this approach also requires systematic single knock-outs for all genes in the network.

The method proposed in [11] has the dual advantages of being very fast to compute and being quite general, as it does not require any assumption of acyclicity of the graph. In addition, as this method provided the best network estimation in the Dialogue for Reverse Engineering Assessments and Methods (DREAM4) *in silico* 100-gene network sub-challenge [13-16], it may be considered as a reference. We note that the method with the best performance for the DREAM4 10-gene network subchallenge was that of [17], based on an automated approach using Petri Nets with Fuzzy Logic; unfortunately, no software is publicly available to implement this method, making it difficult to use in practice.

In this work we propose a novel method in the context of GBNs using a Markov chain Monte Carlo (MCMC) algorithm and Mallows model that is flexible enough to accurately infer causal gene networks from an arbitrary mixture of observational and intervention data, including partial and multiple gene knock-out experiments. As such, the novelty of the proposed method is as follows: 1) it is the only method able to fully make use of all available intervention information, 2) it does not require a systematic intervention experiment to be performed for each gene, and 3) it can deal with sophisticated multiple intervention designs. To benchmark its performance on observational data alone as well as systematic single knock-out data, the proposed method was compared to those of [7] and [11] on simulated data as well as the data from the DREAM4 challenge [13]; in addition, we also consider more complicated simulations based on partial and multiple knock-out designs.

## Methods

### Gaussian Bayesian network framework

Let *G* = (*V*,*E*) be a graph defined by a set of vertices *V* and edges *E*⊂(*V*×*V*). Let the vertices of a graph represent *p* random variables *X*_1_,…,*X*_*p*_. As in the approach of [7], we consider here the framework of causal GBNs, which correspond to Bayesian networks where the nodes have a Gaussian residual distribution and edges represent linear dependencies. In this case, it also follows that the joint distribution of the network is multivariate Gaussian.

In DAGs such as GBNs, we often encounter the presence of Markov equivalence classes, i.e. multiple network structures that yield the same joint distribution; in such cases, observational data alone generally cannot orient edges. For this reason, in many cases the use of intervention data can help overcome this issue, as presented below.

#### Calculation of causal effects

Following an intervention on a given node *X*_*i*_, denoted do(*X*_*i*_ = *x*), we consider the expected value of each other gene in the network via do-calculus as shown in Theorem 3.2.2 (Adjustment for direct causes) in [10]:

$$\begin{array}{l} E(X_{j}|\mathrm{do}(X_{i}=x))\;\\ \;=\;\left\{\begin{array}{c} \;E(X_{j})\;\text{if}\;X_{j}\in \mathrm{pa}(X_{i})\\ \;\int E(X_{j}|x,\mathrm{pa}(X_{i}))\mathbb{P}(\mathrm{pa}(X_{i}))d\mathrm{pa}(X_{i})\;\text{if}\;X_{j}\notin \mathrm{pa}(X_{i}) \end{array}\right)\; \end{array}$$

where pa(*X*_*i*_) represents the parents of node *X*_*i*_. It is important to point out that ℙ(Y|do(X=x)) is different from the conditional probability ℙ(Y|X=x). Using this framework, the total causal effects may be defined as follows:

$$\beta_{\text{ij}}=\frac{\partial }{\mathrm{\partial x}}E(X_{j}|\mathrm{do}(X_{i}=x))$$

and are equal to 0 if *X*_*i*_ is not an ancestor of *X*_*j*_. On the other hand, the direct causal effects (i.e. the edges in the graph) are defined as:

$$\alpha_{\text{ij}}=\frac{\partial }{\mathrm{\partial x}}E(X_{j}|\mathrm{pa}(X_{j}),\mathrm{do}(X_{i}=x)).$$

### Proposed causal inference method

In the GBN framework, when observational data are jointly modeled with intervention data for an arbitrary subset of genes, the network follows a multivariate Gaussian distribution of dimension equal to the number of genes that had no intervention (as the expression value of the gene under intervention is fixed to a given value), and the log-likelihood value can subsequently be calculated for a proposed network.

The calculations in the following section assume that the nodes in the graph have been sorted according to an appropriate causal ordering in the graph such that if *i*<*j*, then *X*_*j*_ is not an ancestor of *X*_*i*_; we note that such an ordering is possible under the assumption of acyclicity of the graph. In practice, of course, it is typically not possible to correctly order nodes in such a way without knowledge of the underlying DAG. For this reason, we aim to explore various network structures based on causal orderings, and to choose among those with the best likelihood value for an arbitrary set of observational and intervention data. The Metropolis-Hastings algorithm [18,19], through the use of a proposal distribution for causal orderings, allows such an exploration to take place and to approach a local maximum of the likelihood.

#### Likelihood calculation

Let *p* be the number of nodes in the graph, *G* the DAG structure and **W** the matrix containing the values for all edges. The nodes are assumed to have been sorted by parental order for *G* and **W**, i.e. if *i*<*j*, then *X*_*j*_ is not an ancestor of *X*_*i*_. This sorting is possible under the assumption of acyclicity and may not necessarily be unique. Under this ordering, **W** is an upper triangular matrix and thus nilpotent. In the GBN framework, it is assumed that each node of *G* has a residual Gaussian distribution, independently from the rest of the network. Let us consider $X_{I}$ with I={1,…,p}, a set of *p* Gaussian random variables defined by:

(1) $$X_{j}=m_{j}+\underset{i\in \text{pa}(j)}{\sum }w_{i,j}X_{i}+\varepsilon_{j}\;\text{with}\;\varepsilon_{j}∼N(0,\sigma_{j}^{2}).$$

We assume that the *ε*_*j*_ are independent, and that *i*∈pa(*j*)⇒*i*<*j* (this assumption is equivalent to assuming that the directed graph obtained using the parental relationships is acyclic). Given the parental structure of the graph, *w*_*i*,*j*_ may only be nonzero on the edge set, (i,j)∈E={i∈pa(j),j∈I}.

Let us now consider the matrix form of Equation (1):

X=m+XW+ε

where **X** = (*X*_1_,…,*X*_*p*_), **m** = (*m*_1_,…,*m*_*p*_), and ***ε*** = (*ε*_1_,…,*ε*_*p*_) are row-vectors of dimension *p*, and **W** = (*w*_*i*,*j*_)_1≤*i*,*j*≤*p*_ is a *p*-dimensional square matrix. By recursively applying this formula and taking advantage of the nilpotence of matrix **W**, we obtain:

X=mL+εL

where **L** = (**I**-**W**)^-1^ = **I** + **W** + … + **W**^*p*-1^. This proves that the model defined in Equation (1) is equivalent to X∼N(μ,Σ) with:

$$\mu =mL\;\text{and}\;\Sigma =L^{T}\text{diag}(\sigma^{2})L=\underset{j\in I}{\sum }\sigma_{j}^{2}L^{T}e_{j}^{T}e_{j}L$$

where **e**_*j*_ is a *p*-dimensional null row-vector except for its *j*^th^ term which is equal to 1, and where ***σ*** = (*σ*_1_,…,*σ*_*p*_) is a row-vector of dimension *p*.

The log-likelihood of the model given *N* observations $x^{k}=(x_{1}^{k},\ldots ,x_{p}^{k})$ (1≤*k*≤*N*) is then:

$$\begin{array}{c} \ell (m,\sigma ,W)=-\frac{\text{Np}}{2}\log(2\pi )-N\underset{j\in I}{\sum }\log(\sigma_{j})\\ \;-\frac{1}{2}\overset{N}{\underset{k=1}{\sum }}\underset{j\in I}{\sum }\frac{1}{\sigma_{j}^{2}}{\left(x_{j}^{k}-x^{k}We_{j}^{T}-m_{j}\right)}^{2}. \end{array}$$

To see this, let us define **A**_*k*_ = (**x**^*k*^-**mL**) ***Σ***^-1^(**x**^*k*^-**mL**)^*T*^ for all *k*. Since ***Σ***^-1^ = (**I**-**W**)diag(1/***σ***^2^)(**I**-**W**)^*T*^ we get:

$$\begin{array}{l} A_{k}=\underset{j\in I}{\sum }\frac{1}{\sigma_{j}^{2}}(x^{k}(I-W)-m)e_{j}^{T}e_{j}{\left(x^{k}(I-W)-m\right)}^{T}\\ \;=\underset{j\in I}{\sum }\frac{1}{\sigma_{j}^{2}}{\left(x_{j}^{k}-x^{k}We_{j}^{T}-m_{j}\right)}^{2}. \end{array}$$

As shown in the Additional file 1, analytical formulae can be obtained for the derivatives with respect to parameters ***θ*** = (**m**, ***σ***,**W**).

The likelihood presented above only takes into account observational data. Let us now consider the case of an arbitrary mixture of observational and intervention data. We assume that we perform an intervention on a subset J⊂I={1,…,p} of variables by artificially fixing the level of the corresponding variables to a value (typically 0 in the case of knock-out experiments): $\mathrm{do}(X_{J}=x_{J})$. The model is then obtained by assuming that all *w*_*i*,*j*_ = 0 for (i,j)∈E and j∈J; we denote the corresponding matrix $W_{J}$. We also assume that the variables *X*_*j*_ for j∈J are fully deterministic. As before, the resulting model is hence Gaussian: $X_{I}|\mathrm{do}(X_{J}=x_{J})∼N(\mu_{J}(x_{J}),\Sigma_{J})$ with

$$\mu_{J}(x_{J})=\nu_{J}(x_{J})L_{J},\;\Sigma_{J}=\underset{j\notin J}{\sum }\sigma_{j}^{2}L_{J}^{T}e_{j}^{T}e_{j}L_{J},$$

where

$$\begin{array}{c} \nu_{J}(x_{J})e_{j}^{T}\\ =\left\{\begin{array}{c} \;x_{j}\;\text{if}\;j\;\in \;J\\ \;m_{j}\;\text{otherwise} \end{array}\right)\;\text{and}\;L_{J}={\left(I\;-\;W_{J}\;\right)}^{-1}\;=\;I\;+\;W_{J}\;+\;\ldots \;+\;W_{J}^{p\;-\;1}. \end{array}$$

For the likelihood calculation, we consider *N* data generated under $x^{k}=(x_{1}^{k},\ldots ,x_{p}^{k})$ (1≤*k*≤*N*) with intervention on $J_{k}$ (where $J_{k}=\emptyset$ means no intervention). We denote by $K_{j}=\{k,j\notin J_{k}\}$, and by $N_{j}=|K_{j}|$ its cardinal. The log-likelihood of the model can then be written as:

(2) $$\begin{array}{c} \ell (m,\sigma ,W)=-\frac{\log(2\pi )}{2}\underset{j}{\sum }N_{j}-\underset{j}{\sum }N_{j}\log(\sigma_{j})\\ \;-\frac{1}{2}\underset{k}{\sum }\underset{j\notin J_{k}}{\sum }\frac{1}{\sigma_{j}^{2}}{\left(x_{j}^{k}-x^{k}We_{j}^{T}-m_{j}\right)}^{2}. \end{array}$$

This is mainly due to the fact that for any intervention set  we have $W_{J}e_{j}^{T}=We_{j}^{T}$ for all j∉J. Considering the derivative with respect to *m*_*j*_ for all *j* such that *N*_*j*_>0, we obtain:

$$m_{j}=\frac{1}{N_{j}}\underset{k\in K_{j}}{\sum }(x_{j}^{k}-x^{k}We_{j}^{T})$$

which can be plugged into the likelihood expression to get:

$$\begin{array}{c} \tilde{\ell }(\sigma ,W)=-\frac{\log(2\pi )}{2}\underset{j}{\sum }N_{j}-\underset{j}{\sum }N_{j}\log(\sigma_{j})\\ \;-\frac{1}{2}\underset{k}{\sum }\underset{j\notin J_{k}}{\sum }\frac{1}{\sigma_{j}^{2}}{\left(y_{j}^{k,j}-y^{k,j}We_{j}^{T}\right)}^{2} \end{array}$$

where for (*k*,*j*) such that $j\notin J_{k}$ we have:

$$y^{k,j}=x^{k}-\frac{1}{N_{j}}\underset{k^{\prime }\in K_{j}}{\sum }x^{k^{\prime }}$$

and **W** can be estimated by solving the following linear system:

(3) $$\underset{i^{\prime },(i^{\prime },j)\in E}{\sum }w_{i^{\prime },j}\underset{k\in K_{j}}{\sum }y_{i}^{k,j}y_{i^{\prime }}^{k,j}=\underset{k\in K_{j}}{\sum }y_{i}^{k,j}y_{j}^{k,j}\;\text{for all}\;\;(i,j)\in \mathrm{E.}$$

Note that the system might be degenerate if the intervention design gives no insight on some parameters. It is hence finally possible to obtain ***σ*** through:

$$\sigma_{j}^{2}=\frac{1}{N_{j}}\underset{k\in K_{j}}{\sum }{\left(y_{j}^{k,j}-y^{k,j}We_{j}^{T}\right)}^{2}.$$

#### Proposed MCMC algorithm

The Metropolis-Hastings algorithm [18,19] is a random walk over *Ω*, the parameter space of the model. It relies on an instrumental probability distribution *Q* which defines the transition from position *X*_*t*_ to a new position *X*. The probability of moving from state *X*_*t*_ to the new state *X* is defined by:M

(4) $$\begin{array}{l} P(X_{t+1}=X|X_{t})=\min\left\{\frac{\pi (X)Q(X_{t},X)}{\pi (X_{t})Q(X,X_{t})},1\right\} \end{array}$$

where *π*(*X*) is the likelihood function.

In order to propose a new causal node ordering $O^{⋆}$ from the previous ordering , we propose to make use of the Mallows model [20]. Briefly, under this model, the density of a proposed causal ordering is defined as follows:

$$\begin{array}{lll} P(O^{⋆}) & =P(O^{⋆}|O,\phi )\; & \;\\ & =\frac{1}{Z}\phi^{d(O^{⋆},O)}\; & \; \end{array}$$

where *ϕ*∈(0,1] is a fixed temperature parameter, *Z* is a normalizing constant, and *d*(·,·) is a dissimilarity measure between  and $O^{⋆}$ based on the number of pairwise ranking disagreements. In addition, we remark that as the temperature parameter *ϕ* approaches zero, the Mallows model approaches a uniform distribution over all causal orderings, and if *ϕ*=1, the model corresponds to a dirac distribution on the reference ordering . In the following, we will use a reparameterization of the temperature coefficient *ϕ* such that *ϕ* = exp(-1/*η*), with *η*>0. Due to the symmetry of *d*, it is clear that $P(O^{⋆}|O,\phi )=P(O|O^{⋆},\phi )$, which allows a simplification of the *Q* terms in the acceptance ratio in Equation (4). We note that a related MCMC approach to explore the space of causal node orderings was recently proposed by [5] in the case of categorical data, making use of an equi-energy sampler.

Proposals for causal node orderings using the aforementioned Mallows model may be obtained by sampling using a repeated insertion model as described in [21]. Based on this new proposal for the node ordering $O^{⋆}$, maximum likelihood estimators may be calculated for the model parameters ***θ*** = (**m**, ***σ***,**W**) using the likelihood described in Equation (2). Subsequently, the Metropolis-Hastings ratio may be calculated and used to determine whether the proposed causal node ordering is accepted or rejected.

R code to implement the proposed MCMC-Mallows algorithm, as well as a sample script providing an example to run the algorithm for a set of simulated data, may be found in Additional files 2 and 3.

## Results and discussion

### Simulation study

**Figure 1:**
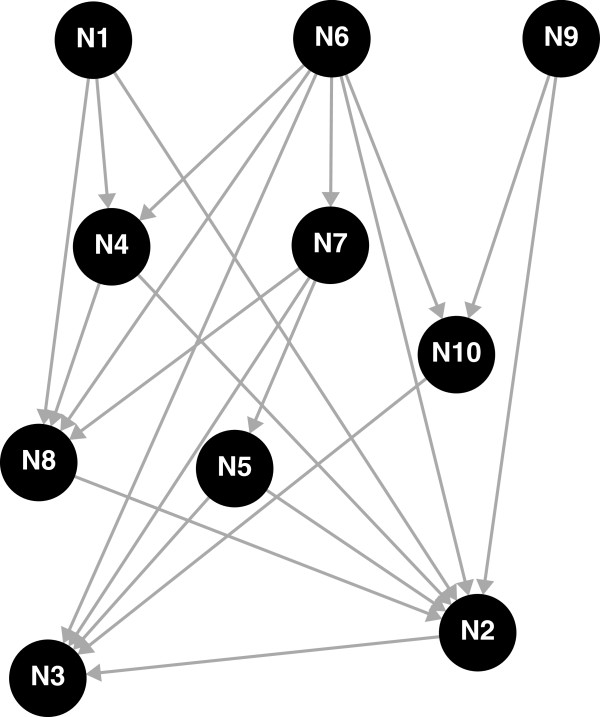
**Graph structure used in simulation study.** Graph structure taken from [9] used for the simulation study for a graph with ten nodes and 21 edges.

Data were simulated under a GBN as in Equation (1) with 10 genes and 21 edges and as described in [9]; the underlying structure is given in Figure 1. For the residual distributions of each gene, we chose 0.5 for the means and three settings for the standard deviations (*σ*=0.01, 0.1 and 0.5), which correspond to small, moderate and large noise for the marginal distributions. Non-zero parameters *w*_*i*,*j*_ were simulated with values drawn uniformly from (-1,-0.25)∪(0.25,1), and for each setting, 100 datasets were generated. The goal was to try to accurately infer the total and direct causal effects among genes.

Several intervention designs were simulated: 1) 20 observational (wild-type) replicates with no interventions, 2) a mixed setting with 10 wild-types and one knock-out per gene, 3) a partial knock-out design with 15 wild-types and one knock-out for five genes: {N1, N4, N6, N7, N9}, 4) a multiple knock-out design with 10 wild types, one knock-out per gene and five double knock-outs: {N1, N5}, {N1, N6}, {N4, N7}, {N6, N9}, and {N7, N10} and 5) a multiple knock-out design as in the previous setting, where all simulated data for three randomly chosen genes were removed (resulting in a set of three hidden variables). Note that we have previously shown [22] that observational data alone (Setting 1 described above) are not informative for the causal node ordering as in such a case, the likelihood is invariant to permutations of the order. Consequently, in this setting node orderings were uniformly sampled rather than using the MCMC-Mallows algorithm; we refer to this strategy as MCMC-uniform.

An MCMC algorithm with Mallows proposal distribution was run to explore the posterior distribution of causal node orderings, as presented in the previous section, with full estimation of ***θ*** = (**m**, ***σ***,**W**) using the maximum likelihood estimators. For the simulations, a small trial run of 1000 iterations was run over a range of possible temperature values *η* (0.2 to 1.5 by 0.1) for the Mallows model, and the value yielding an acceptance rate closest to 30 to 40% [23] was subsequently used for the full run of the MCMC algorithm. In all simulation settings tested here, this value was chosen to be *η* = 0.6 (for *σ* = 0.01 and 0.1) or *η* = 1 (for *σ* = 0.5). As a comparison, we also attempted a trial run of the algorithm using a naive uniform proposal distribution (*η* = 10^10^) in place of the Mallows model, which generally led to acceptance rates of less than 1%. The MCMC-Mallows algorithm was subsequently run for 50,000 iterations, including a burn-in of 5000 iterations and thinning every 50 iterations. We note that due to the analytical maximization step of the likelihood, the method is quite fast and takes only a few minutes to run for each dataset.

In order to benchmark its performance on observational data alone as well as systematic single knock-out data, the proposed algorithm was compared to two previously proposed methods: 1) Pinna [11], which requires a single, systematic knock-out to be performed for every gene, and 2) IDA [7] using the PC-algorithm [2], which only makes use of the observational data. As the PC-algorithm used by [7] provides bounds (*a*,*b*) for the estimated causal effects, we considered two options to facilitate comparisons with the other methods: an “optimistic” calculation, where we use the value max(abs(*a*,*b*)), and a more conservative “pessimistic” strategy, using the value min(abs(*a*,*b*)) if *a* and *b* have the same sign, 0 otherwise.

Finally, several criteria were used to compare the different methods on both total causal effects and direct causal effects: area under the receiver operating characteristic (ROC) curve (AUROC), area under the precision-recall curve (AUPRC), Spearman correlation between true and estimated total or direct causal effects, and the mean squared error (MSE) of estimated total or direct causal effects. Note that the results are calculated for the full **L** = (**I**-**W**)^-1^ (total causal effects) and **W** matrices (direct causal effects) and not just the upper triangular. For the AUROC and AUPRC calculations, positive edges corresponded to (total or direct) causal effects with a nonzero value, and negatives corresponded to (total or direct) causal effects with a null value.

Results for total causal effects are presented in Table 1 for *σ* = 0.1, and in Tables S1 and S2 in Additional file 1 for *σ*=0.01 and 0.5. It can first be noted that results for the IDA method are identical for different levels of variation *σ*; this is due to the fact that it operates on sufficient statistics (correlation matrices) rather than on the data themselves. Similarly, results are identical for the MCMC-uniform method at different levels of *σ* when only observational data are present. Based on observational data only, we note that the proposed algorithm performs as well as the IDA approach; this is unsurprising as both methods are based on GBNs.

**Table 1:** **Comparison of methods for total causal effects for simulated data with moderate variability (*σ=0.1*)**

**Setting**	**Criterion**	**MCMC-Mallows**	**Pinna**	**IDA (opt)**	**IDA (pes)**
	AUROC	0.749 (0.043)	—	0.76 (0.062)	0.643 (0.079)
	AUPRC	0.638 (0.053)	—	0.628 (0.078)	0.527 (0.088)
Observation only	Spearman	0.48 (0.091)	—	0.491 (0.128)	0.254 (0.177)
	MSE	0.056 (0.007)	—	0.182 (0.054)	0.126 (0.034)
	AUROC	0.948 (0.03)	0.825 (0.048)	0.733 (0.068)	0.67 (0.073)
	AUPRC	0.868 (0.042)	0.737 (0.059)	0.569 (0.087)	0.53 (0.091)
Mixed	Spearman	0.696 (0.053)	0.553 (0.097)	0.42 (0.14)	0.318 (0.186)
	MSE	0.026 (0.012)	0.104 (0.011)	0.334 (0.137)	0.196 (0.067)
	AUROC	0.845 (0.059)	0.795 (0.017)	0.736 (0.056)	0.646 (0.085)
	AUPRC	0.734 (0.078)	0.725 (0.038)	0.588 (0.075)	0.514 (0.092)
Partial KO	Spearman	0.587 (0.104)	0.636 (0.034)	0.449 (0.099)	0.285 (0.187)
	MSE	0.035 (0.015)	0.081 (0.008)	0.215 (0.066)	0.146 (0.049)
	AUROC	0.959 (0.016)	0.83 (0.035)	0.733 (0.068)	0.67 (0.073)
	AUPRC	0.886 (0.028)	0.725 (0.039)	0.569 (0.087)	0.53 (0.091)
Multiple KO	Spearman	0.712 (0.028)	0.625 (0.058)	0.42 (0.14)	0.318 (0.186)
	MSE	0.015 (0.006)	0.107 (0.008)	0.334 (0.137)	0.196 (0.067)
	AUROC	0.932 (0.046)	0.574 (0.165)	0.58 (0.145)	0.562 (0.121)
Multiple KO	AUPRC	0.539 (0.078)	0.36 (0.105)	0.353 (0.086)	0.35 (0.08)
(3 hidden genes)	Spearman	0.67 (0.109)	0.037 (0.372)	0.076 (0.316)	0.076 (0.31)
	MSE	0.044 (0.034)	0.15 (0.041)	0.45 (0.225)	0.294 (0.124)

Several intervention designs were simulated: 1) 20 observational (wild-type) replicates with no interventions, 2) mixed setting with 10 wild-types and one knock-out per gene, 3) partial knock-out design with 15 wild-types and one knock-out for five genes {N1, N4, N6, N7, N9}, 4) multiple knock-out design with 10 wild types, one knock-out per gene and five double knock-outs: {N1, N5}, {N1, N6}, {N4, N7}, {N6, N9}, and {N7, N10}, and 5) a multiple knock-out design as in the previous setting, with three hidden variables. Results were averaged over 100 simulations (standard deviations in parentheses): area under the ROC curve (AUROC), area under the precision-recall curve (AUPRC), Spearman correlation between true and estimated total causal effects, and mean squared error (MSE) of estimated total causal effects.

When single knock-outs were simulated (one for each gene) with a large variability (*σ* = 0.5), the IDA [7] approach has slightly more accurate estimation of causal effects than Pinna [11], although we recall that the former method solely makes use of the observational data. On the other hand, when the amount of variability decreases (*σ* = 0.1 and 0.01), the Pinna approach outperforms IDA, even for the optimistic version. In all three settings (*σ* = 0.5, 0.1, 0.01), the proposed MCMC-Mallows algorithm was better able to estimate the causal effects than either Pinna or IDA, as shown by the different criteria presented here. Similar conclusions may be obtained in the context of partial intervention designs. The MCMC-Mallows approach was found to outperform the IDA approach, especially for moderate and low variability. As it requires knock-outs to be performed for all genes in the network, the performance of the Pinna approach suffers when only a subset of interventions are available.

In addition, it was found that considering multiple knock-outs led to an improvement of the estimation of the causal effects over single knock-outs alone. We note that, like the partial knock-out design, this complex intervention design can only be fully accommodated by the proposed MCMC-Mallows method. In this setting, the Pinna method uses only information on the 10 single knock-outs and the IDA approach only the observational data. Finally, in the multiple knock-out setting where data for three genes were hidden, resulting in a set of latent variables, we note that the MCMC-Mallows approach appears to be least affected by the missing information and maintains a satisfactory performance. Similar conclusions may be drawn concerning the comparisons among methods for the direct total causal effects, shown in Table 2 and Tables S3 and S4 in Additional file 1.

**Table 2:** **Comparison of methods for direct causal effects for simulated data with moderate variability (*σ = 0.1*)**

**Setting**	**Criterion**	**MCMC-Mallows**	**Pinna**	**IDA (opt)**	**IDA (pes)**
	AUROC	0.79 (0.041)	—	0.773 (0.064)	0.651 (0.083)
	AUPRC	0.633 (0.061)	—	0.577 (0.085)	0.472 (0.102)
Observation only	Spearman	0.474 (0.094)	—	0.484 (0.122)	0.246 (0.17)
	MSE	0.059 (0.006)	—	0.193 (0.057)	0.138 (0.035)
	AUROC	0.951 (0.03)	0.842 (0.051)	0.746 (0.06)	0.678 (0.073)
	AUPRC	0.841 (0.051)	0.688 (0.084)	0.5 (0.081)	0.465 (0.091)
Mixed	Spearman	0.668 (0.055)	0.534 (0.097)	0.409 (0.132)	0.306 (0.181)
	MSE	0.048 (0.015)	0.107 (0.01)	0.35 (0.131)	0.211 (0.067)
	AUROC	0.871 (0.064)	0.784 (0.018)	0.749 (0.068)	0.655 (0.094)
	AUPRC	0.721 (0.089)	0.663 (0.054)	0.532 (0.088)	0.459 (0.115)
Partial KO	Spearman	0.574 (0.106)	0.606 (0.04)	0.437 (0.104)	0.272 (0.185)
	MSE	0.055 (0.015)	0.088 (0.007)	0.228 (0.068)	0.161 (0.052)
	AUROC	0.962 (0.017)	0.839 (0.032)	0.746 (0.06)	0.678 (0.073)
	AUPRC	0.864 (0.034)	0.69 (0.046)	0.5 (0.081)	0.465 (0.091)
Multiple KO	Spearman	0.683 (0.033)	0.614 (0.051)	0.409 (0.132)	0.306 (0.181)
	MSE	0.038 (0.009)	0.108 (0.008)	0.35 (0.131)	0.211 (0.067)
	AUROC	0.94 (0.045)	0.561 (0.189)	0.576 (0.156)	0.555 (0.133)
Multiple KO	AUPRC	0.483 (0.085)	0.288 (0.107)	0.279 (0.078)	0.276 (0.073)
(3 hidden genes)	Spearman	0.633 (0.106)	0.048 (0.37)	0.07 (0.311)	0.064 (0.305)
	MSE	0.069 (0.048)	0.149 (0.032)	0.454 (0.207)	0.296 (0.109)

Several intervention designs were simulated: 1) 20 observational (wild-type) replicates with no interventions, 2) mixed setting with 10 wild-types and one knock-out per gene, 3) partial knock-out design with 15 wild-types and one knock-out for five genes {N1, N4, N6, N7, N9}, 4) multiple knock-out design with 10 wild types, one knock-out per gene and five double knock-outs: {N1, N5}, {N1, N6}, {N4, N7}, {N6, N9}, and {N7, N10}, and 5) a multiple knock-out design as in the previous setting, with three hidden variables. Results were averaged over 100 simulations (standard deviations in parentheses): area under the ROC curve (AUROC), area under the precision-recall curve (AUPRC), Spearman correlation between true and estimated direct causal effects, and mean squared error (MSE) of estimated direct causal effects.

**Figure 2:**
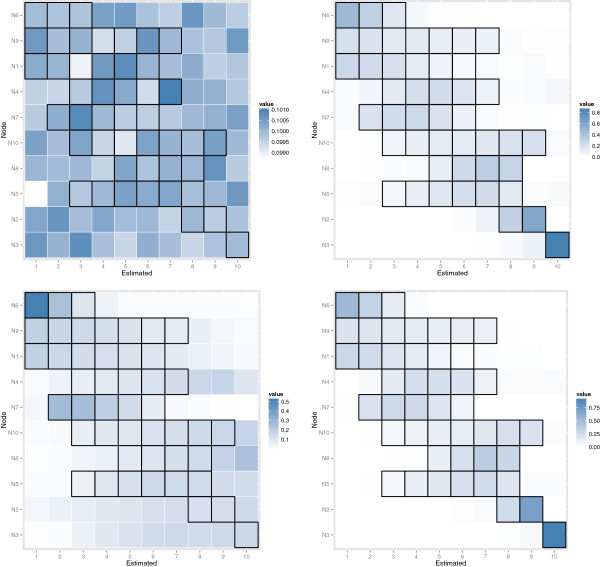
**Posterior distribution of node orders from the MCMC-Mallows approach, averaged over 100 simulations.** Results from simulation setting with *σ* = 0.1: Observations only (top left), complete single knock-outs (top right), partial single knock-outs (bottom left), multiple knock-outs (bottom right). Node labels are included on the vertical axis, estimated positions within causal orderings along the horizontal axis, and the intensity of color of each square corresponds to the average proportion of iterations in which a given node was placed in a given position. As the causal node ordering is not unique for this DAG, true potential positions for each node are outlined in black.

Figure 2 presents the posterior distribution of causal node ordering from the MCMC-Mallows method averaged over 100 simulations for the observation data only (top left), the mixed setting with 10 wild types and one knock-out for each gene (top right), the partial knock-out setting (bottom left), and the multiple knock-out setting (bottom right) for moderately noisy data (*σ* = 0.1). Note that a plot is not included for the hidden variable design, as the true and estimated node orderings are dependent on which three genes are selected to be removed. In these plots, node labels are included on the vertical axis, and estimated positions within orderings along the horizontal axis. Potential orderings for each node within the true graph are highlighted with black outlines; as an example, node N6 could be placed in the first, second, or third position, while node N3 could only be placed in the tenth position in the true graph. The intensity of colors within each box represents the average proportion of iterations in which a node was placed in a particular order. To follow our example, in the mixed setting (top right of Figure 2), on average node N6 was most often placed in the first position, and occasionally positioned second or third, while node N3 was nearly always placed in the last position.

We may remark on several points. First, as shown in the Methods section, it is not possible to estimate the node orders from observational data only. As expected, the node orders were more accurately estimated when a complete knock-out design was considered, with one knock-out for each gene, than for a partial knock-out design. For low to medium variability (*σ*=0.01 and 0.1) the proposed algorithm was able to very accurately estimate the potential node orders for the complete and multiple knock-out designs (see Figures S1–S4 in Additional file 1). Finally, we note that the node ordering is not unique for the DAG considered here, as illustrated by the black squares in Figure 2.

### DREAM data analysis

The proposed MCMC-Mallows algorithm as well as the two previously presented methods [7,11] were applied to data from the DREAM4 challenge, an international competition held yearly to contribute to the development of powerful inference methods [13-16]. In the DREAM4 *in silico* network challenge, network topologies (with feedback loops) were extracted from transcriptional regulatory networks of *E. coli* and *S. cerevisiae*, and data were subsequently simulated and distributed to the participants. The goal was to infer directed regulatory networks from simulated data with either 10 or 100 genes. Based on the considered evaluation criteria (AUROC and AUPRC), the Petri Nets with Fuzzy Logic method [17] and Pinna method [11] were found to be the best performers for the 10-gene and 100-gene network challenges, respectively. In this paper we will focus on the five simulated 10-gene networks and perform inference based on wild type and multifactorial perturbation data (jointly considered to be observational data) as well as knock-out data.

**Figure 3:**
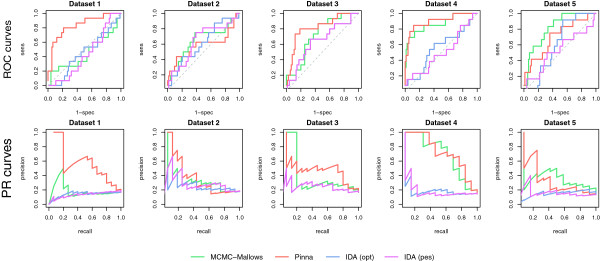
**Comparison of methods on data with a complete design from the DREAM4 challenge.** ROC curves (top) and precision-recall curves (bottom) for the five simulated 10-gene networks of the DREAM4 challenge [13] for the MCMC-Mallows, Pinna, and IDA (optimistic and pessimistic) methods.

Figure 3 presents the ROC curves as well as the precision-recall curves for the different methods in each of the five DREAM4 datasets. Table 3 contains the values for the AUROC and AUPRC for each method on each of the five DREAM4 datasets, as well as the overall DREAM score for each. The overall DREAM score is calculated as the average of global AUROC and AUPR scores, which are calculated across all datasets as the mean of the - log10*p*-values (calculated via permuation tests) for each dataset. As a point of reference, the reported performance of the top-performing method from the DREAM4 challenge, Petri Nets with Fuzzy Logic [17], is also provided; we could not confirm these results as no software is publicly available for its implementation.

**Table 3:** Comparison of methods on complete DREAM4 data

**Criterion**	**Dataset**	**Petri**	**MCMC-**	**Pinna**	**IDA**	**IDA**
		**Nets**	**Mallows**		**(opt)**	**(pes)**
	1	0.972	0.447	0.833	0.448	0.413
	2	0.841	0.647	0.584	0.610	0.641
AUROC	3	0.900	0.717	0.816	0.638	0.638
	4	0.954	0.867	0.899	0.554	0.483
	5	0.928	0.814	0.700	0.599	0.534
	1	0.916	0.183	0.506	0.142	0.133
	2	0.547	0.289	0.331	0.243	0.284
AUPRC	3	0.968	0.340	0.416	0.242	0.242
	4	0.852	0.633	0.664	0.162	0.158
	5	0.761	0.308	0.278	0.146	0.156
DREAM score	overall	7.127	2.579	3.563	0.735	0.723

Area under the ROC curve (AUROC), area under the precision-recall curve (AUPRC), and DREAM score for each of the five DREAM4 datasets for the Petri Nets [17], MCMC-Mallows, Pinna *et al.*, and IDA (optimistic and pessimistic) methods. Results for the Petri Nets method [17] and evaluation scripts for the overall DREAM score were obtained from the DREAM4 evaluation page, located at http://wiki.c2b2.columbia.edu/dream/results/DREAM4.

It can first be observed that the IDA [7], whether optimistic or pessimistic versions of the causal effects estimations are used, performs the worst; this is unsurprising, as it only makes use of the observational data. On the other hand, the proposed MCMC-Mallows method compares quite well to the Pinna approach, except for the first data set where Pinna clearly outperforms the others. We note that the simulated intervention setting was well adapted to the Pinna method, as one knock-out was available for each gene; in addition, we note that as the MCMC-Mallows and IDA methods are based on a causal Bayesian network framework, feedback loops in the network cannot be modeled due to the assumption of acyclicity in the graph. Finally, it can be seen that the Petri Nets method of [17] significantly outperforms the other methods on these data; however, we recall that the major contribution of our proposed MCMC-Mallows approach is not its ability to best model complete, single knock-out intervention designs but rather its unique flexibility to accommodate more complex or incomplete intervention designs, as we demonstrate in the following.

**Figure 4:**
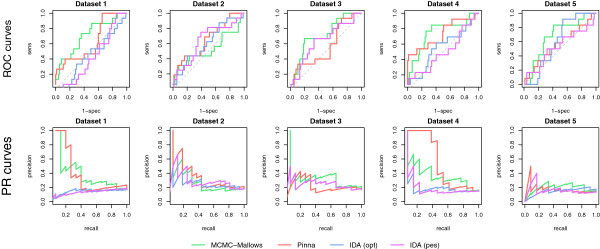
**Comparison of methods on data with a partial design from the DREAM4 challenge.** ROC curves (top) and precision-recall curves (bottom) for the five simulated 10-gene networks of the DREAM4 challenge [13], where for each dataset five knock-outs were removed at random, for the MCMC-Mallows, Pinna, and IDA (optimistic and pessimistic) methods.

To assess the performance of each of the methods on the DREAM4 data with only an incomplete set of gene knock-out experiments (similar to the partial knock-out simulation above), we remove half of the knock-out experiments (chosen at random) from each dataset. Figure 4 presents the ROC and precision-recall curves for this partial knock-out setting and Table 4 provides the AUROC, AUPRC, and overall DREAM scores. As no software is publicly available to implement the Petri Nets approach, no results for this method may be obtained in this context. The performance of IDA is identical in this setting to that of the full data, as it uses the observational data alone. The loss of information as compared to the complete data is reflected in the lower overall DREAM scores for both the MCMC-Mallows and Pinna approaches; we note that in nearly all cases (with the exception of the second dataset), the Pinna method is adversely affected by the loss of intervention data as compared to the previous results. On the other hand, the MCMC-Mallows appears to be the least adversely affected by the incomplete design and maintains a similar performance to the complete design. As such, although the complete intervention design clearly yields more information about causal effects among genes, the MCMC-Mallows approach appears to be best able to extract pertinent information when only partial intervention designs are available.

**Table 4:** Comparison of methods on partial DREAM4 data

**Criterion**	**Dataset**	**MCMC-**	**Pinna**	**IDA**	**IDA**
		**Mallows**		**(opt)**	**(pes)**
	1	0.708	0.555	0.448	0.413
	2	0.525	0.637	0.610	0.641
AUROC	3	0.711	0.498	0.638	0.638
	4	0.748	0.682	0.554	0.483
	5	0.676	0.565	0.599	0.534
	1	0.344	0.346	0.142	0.133
	2	0.240	0.306	0.243	0.284
AUPRC	3	0.271	0.214	0.242	0.242
	4	0.322	0.494	0.162	0.158
	5	0.194	0.168	0.146	0.156
DREAM score	overall	1.844	1.450	0.735	0.723

Area under the ROC curve (AUROC), area under the precision-recall curve (AUPRC), and DREAM score for each of the five DREAM4 partial datasets, where only five of the single-gene knock-outs are included, for the MCMC-Mallows, Pinna *et al.*, and IDA (optimistic and pessimistic) methods. Results for the Petri Nets method [17] are not provided as no software is publicly available to implement this approach. Evaluation scripts for the overall DREAM score were obtained from the DREAM4 evaluation page, located at http://wiki.c2b2.columbia.edu/dream/results/DREAM4.

## Conclusions

In this paper we proposed a flexible and powerful approach for joint causal network inference from both observational and intervention data, using an MCMC algorithm and Mallows model. The computational efficiency of the method is very much improved by the analytical maximization step of the likelihood.

In the simulation study presented above, the proposed MCMC-Mallows algorithm was found to perform better than Pinna [11] and IDA [7] in terms of accuracy of estimation of the causal effects, as evidenced by the tendancy to have larger AUROC, larger Spearman correlation coefficients and smaller MSE than the other approaches. Additionally, our simulations demonstrated that multiple knock-out designs contributed valuable additional information for causal network inference beyond single knock-outs; we therefore anticipate that the need for methods able to accommodate complex intervention designs will only increase as such data become more common. The results for the complete DREAM4 data are somewhat inconclusive, with Pinna performing best on two datasets, MCMC-Mallows best on two others, and nearly equivalent performance on the last; in addition, all methods considered here performed considerably worse than the winning Petri Nets method of [17] on the complete set of data. We note that the DREAM networks, like many real biological networks, contain feedback loops that cannot be modeled by methods based on causal Bayesian networks such as MCMC-Mallows and IDA. However, despite this limitation, the results of the partial design for DREAM4 data demonstrate that the MCMC-Mallows method is best able to accommodate complex intervention designs, including partial gene knock-outs. In fact, the novelty of the MCMC-Mallows approach, and the primary contribution of this work, lies in its flexibility to model arbitrary single, multiple, and partial knock-out designs.

In its present form, the proposed algorithm is not applicable to large-scale networks made up of several hundreds of nodes. Due to the curse of dimensionality, the size of the search space of causal node orderings explodes in dimension as the number of nodes increases, meaning that alternative MCMC samplers, such as parallel tempering, may be better suited to such situations. In addition, the resolution of the linear system in Equation (3) needed for the likelihood calculation has complexity *O*(*p*^6^) when no sparsity constraints are included for matrix **W**. As such, the generalization of the proposed algorithm to a *p*>>*n* situation will require the addition of a ridge or Lasso penalty, as recently proposed by [24], as well as a modification of the proposal distribution and sampling strategy. The current algorithm is fully compatible with such extensions and this will be the focus of our research in the near future.

The choice of optimal experimental knock-out designs is an important issue for causal inference and merits further attention. Hauser and Bühlmann [25] recently proposed two strategies for the choice of optimal interventions. The first is a greedy approach using single-vertex interventions that maximizes the number of edges that can be oriented after each intervention; the second yields a minimum set of targets of arbitrary size that guarantee full identifiability. However, alternative approaches could be envisaged in future research. In particular, recall that in the GBN framework, the likelihood associated to the multivariate Gaussian distribution of the network can be explicitly written as presented in this work. The choice of optimal knock-outs to be performed to improve and validate the causal inference can then rely on the evaluation of the amount of information contributed by each possible intervention, which can for example be obtained by the Fisher information. Its calculation requires the derivation of the likelihood function, which is not trivial but has already been derived in [22]. We anticipate that this issue will remain an interesting challenge for future research.

## Competing interests

The authors declare that they have no competing interests.

## Authors’ contributions

AR participated in the design of the study, performed simulations and data analyses, and helped draft the manuscript. FJ participated in the design of the study and drafted the manuscript. GN designed the study, performed the analytical likelihood calculations and helped draft the manuscript. All authors read and approved the final manuscript.

## Supplementary Material

Additional file 1 **Supplementary materials.** This file contains details for calculations as well as additional results from the simulation study presented in the main paper. Click here for file

Additional file 2 **R code to implement MCMC-Mallows approach.** This file contains the R code to implement the proposed MCMC-Mallows approach. Click here for file

Additional file 3 **Example R code to run MCMC-Mallows approach.** This file contains the R code to run the proposed MCMC-Mallows approach for a set of simulated data. Click here for file
