# Supplementary material for: Joint estimation of causal effects from observational and intervention gene expression data
Source: BMC Syst Biol. 2013 Oct 31;7:111. doi: 10.1186/1752-0509-7-111 (PMC3834107; doi:10.1186/1752-0509-7-111)
Supplement: Additional file 1 — Supplementary materials. This file contains details for calculations as well as additional results from the simulation study presented in the main paper. [file 1752-0509-7-111-S1.pdf]

Supplementary Materials: Joint estimation of causal effects from observational and intervention gene expression data

A. Rau*† F. Jaffrézic*† G. Nuel‡

1 Additional details of analytical formulae for maximum likelihood parameter estimation for observational data

The log-likelihood of the model given N observations $x^k = (x_1^k, \ldots, x_p^k)$ $(1 \leqslant k \leqslant N)$ is:

$$\ell(\mathbf{m}, \boldsymbol{\sigma}, \mathbf{W}) = -\frac{Np}{2}\log(2\pi) - N\sum_{j\in\mathcal{I}}\log(\sigma_j) - \frac{1}{2}\sum_{k=1}^{N}\sum_{j\in\mathcal{I}}\frac{1}{\sigma_j^2}(x_j^k - \mathbf{x}^k\mathbf{W}\mathbf{e}_j^T - m_j)^2. \quad (1)$$

Derivatives with respect to $\mathbf{m}$

$$\frac{\partial \ell}{\partial m_j}(\boldsymbol{\theta}) = \frac{1}{\sigma_j^2}\sum_{k=1}^{N}(x_j^k - \mathbf{x}^k\mathbf{W}\mathbf{e}_j^T - m_j)$$

The maximization of $\ell(\boldsymbol{\theta})$ in $\mathbf{m}$ for a fixed $\mathbf{W}$ does not depend on $\boldsymbol{\sigma}$ and is given by:

$$m_j = \frac{1}{N}\sum_{k=1}^{N}(x_j^k - \mathbf{x}^k\mathbf{W}\mathbf{e}_j^T)$$

by replacing m_j by this formula in Eq. 1 we get an expression of the likelihood free of the parameter $\mathbf{m}$:

$$\tilde{\ell}(\boldsymbol{\sigma}, \mathbf{W}) = -\frac{Np}{2}\log(2\pi) - N\sum_{j\in\mathcal{I}}\log(\sigma_j) - \frac{1}{2}\sum_{k=1}^{N}\sum_{j\in\mathcal{I}}\frac{1}{\sigma_j^2}(y_j^k - \mathbf{y}^k\mathbf{W}\mathbf{e}_j^T)^2$$

with $y_j^k = x_j^k - 1/N\sum_{k'} x_j^{k'}$ for all k, j.

Derivatives with respect to $\mathbf{W}$

$$\frac{\partial \ell}{\partial w_{i,j}}(\boldsymbol{\theta}) = \frac{1}{\sigma_j^2}\sum_{k=1}^{N} y_i^k(y_j^k - \mathbf{y}^k\mathbf{W}\mathbf{e}_j^T).$$

*INRA, UMR1313 Génétique animale et biologie intégrative, 78352 Jouy-en Josas, France
†AgroParisTech, UMR1313 Génétique animale et biologie intégrative, 75231 Paris 05, France
‡MAP5, UMR CNRS 8145, University Paris Descartes, 45 rue des Saints-Pères, F-75006 Paris - France

Proof:

$$\sum_{j'\in\mathcal{I}} \frac{1}{\sigma_{j'}^2}(y_{j'}^k - \mathbf{y}^k\mathbf{W}\mathbf{e}_{j'}^T)(\underbrace{\mathbf{y}^k\mathbf{e}_i^T}_{\mathbf{y}_i^k}\underbrace{\mathbf{e}_j\mathbf{e}_{j'}^T}_{\mathbb{1}_{j'=j}}) = \frac{y_j^k}{\sigma_j^2}(y_j^k - \mathbf{y}^k\mathbf{W}\mathbf{e}_j^T)$$

The maximization of $\tilde{\ell}(\boldsymbol{\sigma}, \mathbf{W})$ in $\mathbf{W}$ can then be done independently from $\boldsymbol{\sigma}$ by solving for all $(i, j) \in \mathcal{E}$:

$$\sum_{k=1}^{N} y_i^k \mathbf{y}^k \mathbf{W}\mathbf{e}_j^T = \sum_{k=1}^{N} y_i^k y_i^k$$

hence using

$$\mathbf{W} = \sum_{(i',j')\in\mathcal{E}} w_{i',j'}\mathbf{e}_{i'}^T\mathbf{e}_{j'} \Rightarrow \mathbf{y}^k\mathbf{W}\mathbf{e}_j^T = \sum_{i',(i',j)\in\mathcal{E}} w_{i',j}y_{i'}^k$$

so that we get:

$$\sum_{i',(i',j)\in\mathcal{E}} w_{i',j}\sum_{k=1}^{N} y_i^k y_{i'}^k = \sum_{k=1}^{N} y_i^k y_j^k \quad \text{for all } (i,j) \in \mathcal{E}$$

Derivatives with respect to $\boldsymbol{\sigma}$

$$\frac{\partial \ell}{\partial \sigma_j}(\boldsymbol{\theta}) = -\frac{N}{\sigma_j} + \frac{1}{\sigma_j^3}\sum_{k=1}^{N}(y_j^k - \mathbf{y}^k\mathbf{W}\mathbf{e}_j^T)^2$$

The maximization of $\tilde{\ell}(\boldsymbol{\sigma}, \mathbf{W})$ in $\boldsymbol{\sigma}$ when $\mathbf{m}$ is fixed is then given by:

$$\sigma_j^2 = \frac{1}{N}\sum_{k=1}^{N}(y_j^k - \mathbf{y}^k\mathbf{W}\mathbf{e}_j^T)^2$$

2 Comparison of methods for total causal effects for simulated data with large variability ($\sigma = 0.5$) and small variability ($\sigma = 0.01$)

Setting	Criterion	MCMC-Mallows	Pinna	IDA (opt)	IDA (pes)
Observation only	AUROC	0.749 (0.043)	—	0.76 (0.062)	0.643 (0.079)
	AUPRC	0.638 (0.053)	—	0.628 (0.078)	0.527 (0.088)
	Spearman	0.48 (0.091)	—	0.491 (0.128)	0.254 (0.177)
	MSE	0.056 (0.007)	—	0.182 (0.054)	0.126 (0.034)
Mixed	AUROC	0.791 (0.09)	0.625 (0.064)	0.733 (0.068)	0.67 (0.073)
	AUPRC	0.654 (0.116)	0.43 (0.069)	0.569 (0.087)	0.53 (0.091)
	Spearman	0.505 (0.145)	0.181 (0.159)	0.42 (0.14)	0.318 (0.186)
	MSE	0.069 (0.027)	0.553 (0.13)	0.334 (0.137)	0.196 (0.067)
Partial KO	AUROC	0.732 (0.072)	0.731 (0.027)	0.736 (0.056)	0.646 (0.085)
	AUPRC	0.595 (0.097)	0.545 (0.066)	0.588 (0.075)	0.514 (0.092)
	Spearman	0.431 (0.149)	0.485 (0.071)	0.449 (0.099)	0.285 (0.187)
	MSE	0.063 (0.02)	0.272 (0.073)	0.215 (0.066)	0.146 (0.049)
Multiple KO	AUROC	0.819 (0.09)	0.613 (0.07)	0.733 (0.068)	0.67 (0.073)
	AUPRC	0.7 (0.109)	0.407 (0.077)	0.569 (0.087)	0.53 (0.091)
	Spearman	0.542 (0.128)	0.162 (0.169)	0.42 (0.14)	0.318 (0.186)
	MSE	0.054 (0.022)	0.399 (0.1)	0.334 (0.137)	0.196 (0.067)
Multiple KO (3 hidden genes)	AUROC	0.795 (0.099)	0.543 (0.118)	0.58 (0.145)	0.562 (0.121)
	AUPRC	0.463 (0.072)	0.333 (0.059)	0.353 (0.086)	0.35 (0.08)
	Spearman	0.512 (0.203)	-0.016 (0.221)	0.076 (0.316)	0.076 (0.31)
	MSE	0.056 (0.029)	0.409 (0.15)	0.45 (0.225)	0.294 (0.124)

Supplementary Table 1: $\sigma = 0.5$**, total causal effects**. Several intervention designs were simulated: 1) 20 observational (wild-type) replicates with no interventions, 2) mixed setting with 10 wild-types and one knock-out per gene, 3) partial knock-out design with 15 wild-types and one knock-out for five genes {N1, N4, N6, N7, N9}, 4) multiple knock-out design with 10 wild types, one knock-out per gene and five double knock-outs: {N1, N5}, {N1, N6}, {N4, N7}, {N6, N9}, and {N7, N10}, and 5) a multiple knock-out design as in the previous setting, with three hidden variables. Results were averaged over 100 simulations (standard deviations in parentheses): area under the ROC curve (AUROC), area under the precision-recall curve (AUPRC), Spearman correlation between true and estimated total causal effects, and mean squared error (MSE) of estimated total causal effects.

Setting	Criterion	MCMC-Mallows	Pinna	IDA (opt)	IDA (pes)
Observation only	AUROC	0.749 (0.043)	—	0.76 (0.062)	0.643 (0.079)
	AUPRC	0.638 (0.053)	—	0.628 (0.078)	0.527 (0.088)
	Spearman	0.48 (0.091)	—	0.491 (0.128)	0.254 (0.177)
	MSE	0.056 (0.007)	—	0.182 (0.054)	0.126 (0.034)
Mixed	AUROC	0.983 (0.015)	0.945 (0.026)	0.733 (0.068)	0.67 (0.073)
	AUPRC	0.933 (0.017)	0.902 (0.023)	0.569 (0.087)	0.53 (0.091)
	Spearman	0.744 (0.027)	0.693 (0.046)	0.42 (0.14)	0.318 (0.186)
	MSE	0.001 (0.001)	0.088 (0.001)	0.334 (0.137)	0.196 (0.067)
Partial KO	AUROC	0.904 (0.032)	0.829 (0.008)	0.736 (0.056)	0.646 (0.085)
	AUPRC	0.798 (0.058)	0.803 (0.013)	0.588 (0.075)	0.514 (0.092)
	Spearman	0.645 (0.035)	0.689 (0.017)	0.449 (0.099)	0.285 (0.187)
	MSE	0.016 (0.009)	0.073 (0.001)	0.215 (0.066)	0.146 (0.049)
Multiple KO	AUROC	0.986 (0.007)	0.896 (0.009)	0.733 (0.068)	0.67 (0.073)
	AUPRC	0.937 (0.012)	0.792 (0.006)	0.569 (0.087)	0.53 (0.091)
	Spearman	0.751 (0.015)	0.691 (0.004)	0.42 (0.14)	0.318 (0.186)
	MSE	0.001 (0.001)	0.097 (0.001)	0.334 (0.137)	0.196 (0.067)
Multiple KO (3 hidden genes)	AUROC	0.948 (0.04)	0.617 (0.192)	0.58 (0.145)	0.562 (0.121)
	AUPRC	0.539 (0.082)	0.373 (0.125)	0.353 (0.086)	0.35 (0.08)
	Spearman	0.675 (0.118)	0.096 (0.422)	0.076 (0.316)	0.076 (0.31)
	MSE	0.078 (0.077)	0.145 (0.041)	0.45 (0.225)	0.294 (0.124)

Supplementary Table 2: $\sigma = 0.01$**, total causal effects**. Several intervention designs were simulated: 1) 20 observational (wild-type) replicates with no interventions, 2) mixed setting with 10 wild-types and one knock-out per gene, 3) partial knock-out design with 15 wild-types and one knock-out for five genes {N1, N4, N6, N7, N9}, 4) multiple knock-out design with 10 wild types, one knock-out per gene and five double knock-outs: {N1, N5}, {N1, N6}, {N4, N7}, {N6, N9}, and {N7, N10}, and 5) a multiple knock-out design as in the previous setting, with three hidden variables. Results were averaged over 100 simulations (standard deviations in parentheses): area under the ROC curve (AUROC), area under the precision-recall curve (AUPRC), Spearman correlation between true and estimated total causal effects, and mean squared error (MSE) of estimated total causal effects.

3 Comparison of methods for direct causal effects for simulated data with large ($\sigma = 0.5$), moderate ($\sigma = 0.1$) and small variability ($\sigma = 0.01$)

Setting	Criterion	MCMC-Mallows	Pinna	IDA (opt)	IDA (pes)
Observation only	AUROC	0.79 (0.041)	—	0.773 (0.064)	0.651 (0.083)
	AUPRC	0.633 (0.061)	—	0.577 (0.085)	0.472 (0.102)
	Spearman	0.474 (0.094)	—	0.484 (0.122)	0.246 (0.17)
	MSE	0.059 (0.006)	—	0.193 (0.057)	0.138 (0.035)
Mixed	AUROC	0.813 (0.085)	0.618 (0.077)	0.746 (0.06)	0.678 (0.073)
	AUPRC	0.617 (0.121)	0.345 (0.078)	0.5 (0.081)	0.465 (0.091)
	Spearman	0.49 (0.139)	0.17 (0.148)	0.409 (0.132)	0.306 (0.181)
	MSE	0.077 (0.024)	0.549 (0.128)	0.35 (0.131)	0.211 (0.067)
Partial KO	AUROC	0.768 (0.076)	0.702 (0.03)	0.749 (0.068)	0.655 (0.094)
	AUPRC	0.57 (0.111)	0.422 (0.073)	0.532 (0.088)	0.459 (0.115)
	Spearman	0.424 (0.147)	0.457 (0.074)	0.437 (0.104)	0.272 (0.185)
	MSE	0.069 (0.017)	0.278 (0.074)	0.228 (0.068)	0.161 (0.052)
Multiple KO	AUROC	0.843 (0.08)	0.614 (0.075)	0.746 (0.06)	0.678 (0.073)
	AUPRC	0.679 (0.112)	0.332 (0.079)	0.5 (0.081)	0.465 (0.091)
	Spearman	0.53 (0.121)	0.158 (0.166)	0.409 (0.132)	0.306 (0.181)
	MSE	0.066 (0.018)	0.395 (0.098)	0.35 (0.131)	0.211 (0.067)
Multiple KO (3 hidden genes)	AUROC	0.825 (0.096)	0.532 (0.126)	0.576 (0.156)	0.555 (0.133)
	AUPRC	0.408 (0.08)	0.257 (0.055)	0.279 (0.078)	0.276 (0.073)
	Spearman	0.493 (0.194)	-0.01 (0.216)	0.07 (0.311)	0.064 (0.305)
	MSE	0.069 (0.029)	0.407 (0.15)	0.454 (0.207)	0.296 (0.109)

Supplementary Table 3: $\sigma = 0.5$**, direct causal effects**. Several intervention designs were simulated: 1) 20 observational (wild-type) replicates with no interventions, 2) mixed setting with 10 wild-types and one knock-out per gene, 3) partial knock-out design with 15 wild-types and one knock-out for five genes {N1, N4, N6, N7, N9}, 4) multiple knock-out design with 10 wild types, one knock-out per gene and five double knock-outs: {N1, N5}, {N1, N6}, {N4, N7}, {N6, N9}, and {N7, N10}, and 5) a multiple knock-out design as in the previous setting, with three hidden variables. Results were averaged over 100 simulations (standard deviations in parentheses): area under the ROC curve (AUROC), area under the precision-recall curve (AUPRC), Spearman correlation between true and estimated direct causal effects, and mean squared error (MSE) of estimated direct causal effects.

Setting	Criterion	MCMC-Mallows	Pinna	IDA (opt)	IDA (pes)
Observation only	AUROC	0.79 (0.041)	—	0.773 (0.064)	0.651 (0.083)
	AUPRC	0.633 (0.061)	—	0.577 (0.085)	0.472 (0.102)
	Spearman	0.474 (0.094)	—	0.484 (0.122)	0.246 (0.17)
	MSE	0.059 (0.006)	—	0.193 (0.057)	0.138 (0.035)
Mixed	AUROC	0.983 (0.008)	0.95 (0.021)	0.746 (0.06)	0.678 (0.073)
	AUPRC	0.911 (0.01)	0.84 (0.021)	0.5 (0.081)	0.465 (0.091)
	Spearman	0.724 (0.018)	0.691 (0.034)	0.409 (0.132)	0.306 (0.181)
	MSE	0.023 (0.002)	0.092 (0.001)	0.35 (0.131)	0.211 (0.067)
Partial KO	AUROC	0.929 (0.023)	0.815 (0.007)	0.749 (0.068)	0.655 (0.094)
	AUPRC	0.802 (0.056)	0.748 (0.011)	0.532 (0.088)	0.459 (0.115)
	Spearman	0.644 (0.031)	0.666 (0.011)	0.437 (0.104)	0.272 (0.185)
	MSE	0.038 (0.009)	0.079 (0.001)	0.228 (0.068)	0.161 (0.052)
Multiple KO	AUROC	0.984 (0.005)	0.882 (0.01)	0.746 (0.06)	0.678 (0.073)
	AUPRC	0.912 (0.008)	0.756 (0.008)	0.5 (0.081)	0.465 (0.091)
	Spearman	0.727 (0.011)	0.66 (0.004)	0.409 (0.132)	0.306 (0.181)
	MSE	0.023 (0.001)	0.099 (0.001)	0.35 (0.131)	0.211 (0.067)
Multiple KO (3 hidden genes)	AUROC	0.944 (0.044)	0.591 (0.204)	0.576 (0.156)	0.555 (0.133)
	AUPRC	0.47 (0.095)	0.296 (0.118)	0.279 (0.078)	0.276 (0.073)
	Spearman	0.642 (0.111)	0.087 (0.408)	0.07 (0.311)	0.064 (0.305)
	MSE	0.104 (0.084)	0.142 (0.029)	0.454 (0.207)	0.296 (0.109)

Supplementary Table 4: $\sigma = 0.01$**, direct causal effects**. Several intervention designs were simulated: 1) 20 observational (wild-type) replicates with no interventions, 2) mixed setting with 10 wild-types and one knock-out per gene, 3) partial knock-out design with 15 wild-types and one knock-out for five genes {N1, N4, N6, N7, N9}, 4) multiple knock-out design with 10 wild types, one knock-out per gene and five double knock-outs: {N1, N5}, {N1, N6}, {N4, N7}, {N6, N9}, and {N7, N10}, and 5) a multiple knock-out design as in the previous setting, with three hidden variables. Results were averaged over 100 simulations (standard deviations in parentheses): area under the ROC curve (AUROC), area under the precision-recall curve (AUPRC), Spearman correlation between true and estimated direct causal effects, and mean squared error (MSE) of estimated direct causal effects.

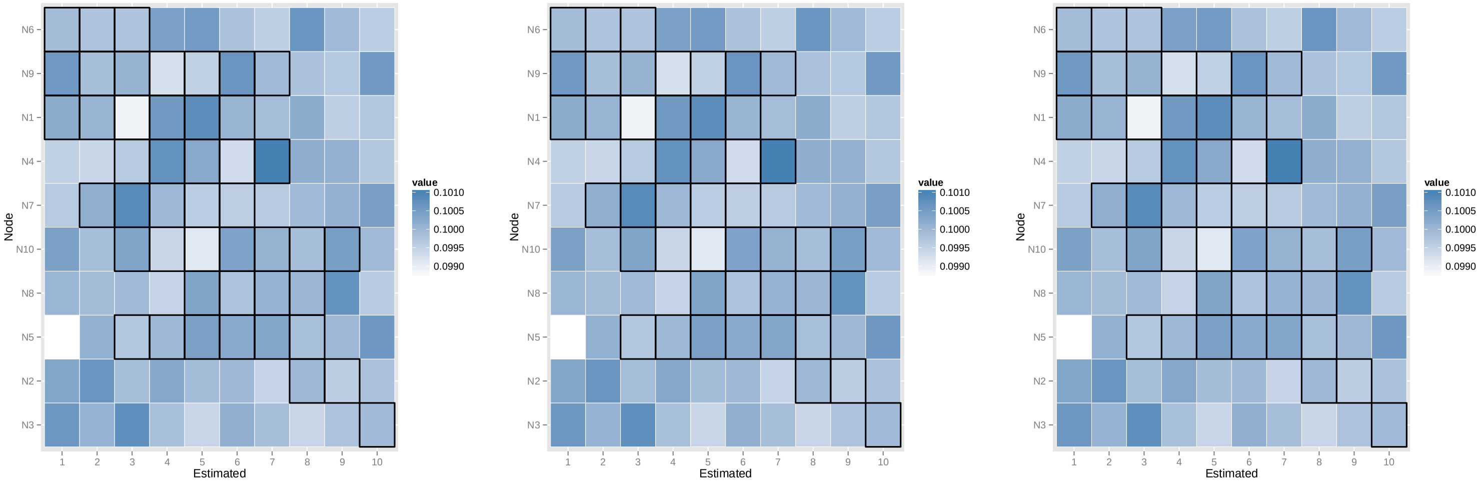

Supplementary Figure 1: $\sigma = 0.5$ **(left),** $\sigma = 0.1$ **(middle),** $\sigma = 0.01$ **(right), observation only setting**. Posterior distribution of node orders, averaged over 100 simulations. Node labels are included on the vertical axis, estimated positions within causal orderings along the horizontal axis, and the intensity of color of each square corresponds to the average proportion of iterations in which a given node was placed in a given position. As the causal node ordering is not unique for this DAG, true potential positions for each node are outlined in black.

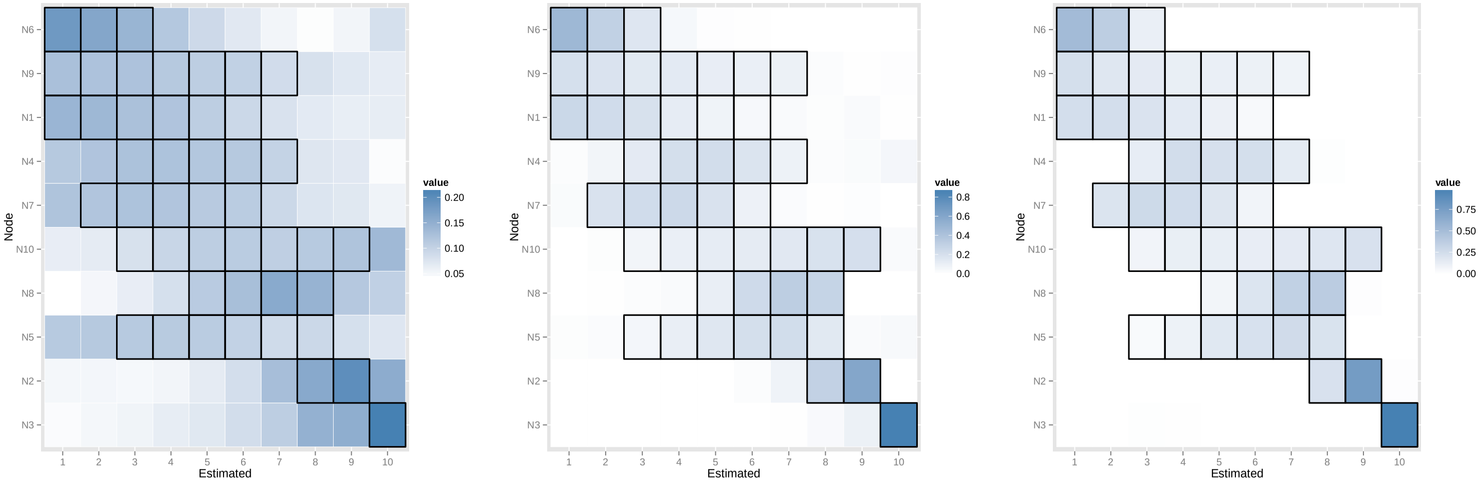

Supplementary Figure 2: $\sigma = 0.5$ **(left),** $\sigma = 0.1$ **(middle),** $\sigma = 0.01$ **(right), mixed setting**. Posterior distribution of node orders, averaged over 100 simulations. Node labels are included on the vertical axis, estimated positions within causal orderings along the horizontal axis, and the intensity of color of each square corresponds to the average proportion of iterations in which a given node was placed in a given position. As the causal node ordering is not unique for this DAG, true potential positions for each node are outlined in black.

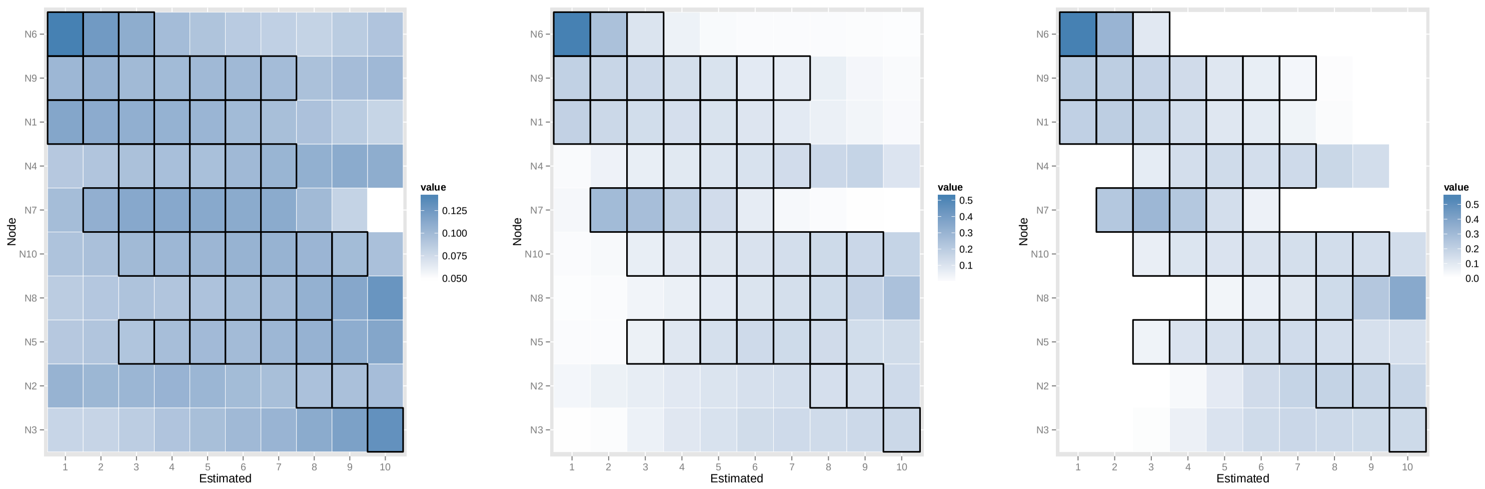

Supplementary Figure 3: $\sigma = 0.5$ **(left),** $\sigma = 0.1$ **(middle),** $\sigma = 0.01$ **(right), partial KO setting**. Posterior distribution of node orders, averaged over 100 simulations. Node labels are included on the vertical axis, estimated positions within causal orderings along the horizontal axis, and the intensity of color of each square corresponds to the average proportion of iterations in which a given node was placed in a given position. As the causal node ordering is not unique for this DAG, true potential positions for each node are outlined in black.

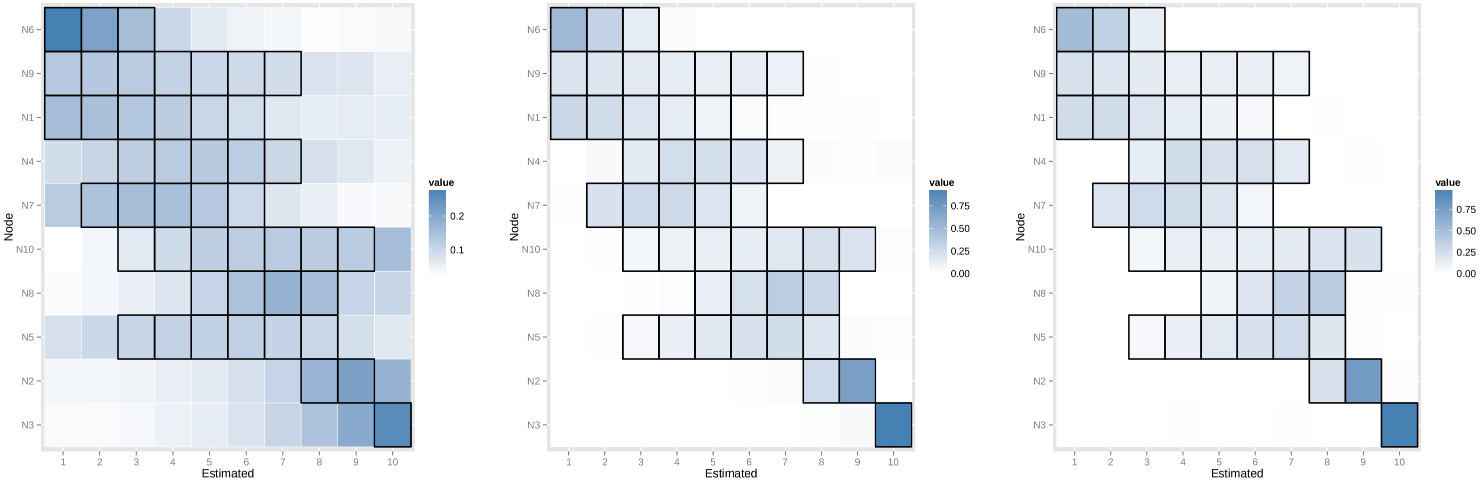

Supplementary Figure 4: $\sigma = 0.5$ **(left),** $\sigma = 0.1$ **(middle),** $\sigma = 0.01$ **(right), multiple KO setting**. Posterior distribution of node orders, averaged over 100 simulations. Node labels are included on the vertical axis, estimated positions within causal orderings along the horizontal axis, and the intensity of color of each square corresponds to the average proportion of iterations in which a given node was placed in a given position. As the causal node ordering is not unique for this DAG, true potential positions for each node are outlined in black.
